# Tuberculous Distal Biceps Tendon Rupture: Case Report and Review of the Literature

**DOI:** 10.1155/2018/6374784

**Published:** 2018-10-25

**Authors:** Artit Boonrod, Hiroyuki Sugaya, Norimasa Takahashi, Arunnit Boonrod, Chat Sumananont

**Affiliations:** ^1^Department of Orthopaedics, Faculty of Medicine, Khon Kaen University, Khon Kaen, Thailand; ^2^Sports Medicine and Joint Center, Funabashi Orthopaedic Hospital, Funabashi, Japan; ^3^Department of Radiology, Faculty of Medicine, Khon Kaen University, Khon Kaen, Thailand

## Abstract

Tuberculous distal biceps tendon rupture is a rare condition in orthopedics. Musculoskeletal tuberculosis usually presents with bursitis, synovitis, myositis, and osteomyelitis, conditions which demonstrate an excellent response to antituberculosis chemotherapy. Tendon rupture is often associated with delayed diagnosis and treatment. We report a rare manifestation of musculoskeletal tuberculosis in the distal biceps tendon with delayed diagnosis.

## 1. Introduction

Tenosynovitis and bursitis occur in about 2% of all extraspinal musculoskeletal tuberculosis cases. Tuberculosis involving the biceps tendon has been reported, but the tendons were still intact and could be treated with drugs and surgical debridement [1, 2]. We report here an uncommon example of complete distal biceps tendon rupture due to disseminated tuberculosis.

## 2. Case Report

A 39-year-old Thai male patient presented with progressive pain and swelling of seven-month duration over the antecubital fossa of the right elbow. Initially, there was only slight swelling. Three months later, he complained of dull pain. The patient went to a private clinic where the diagnosis was distal biceps tendinitis. The first doctor gave a local steroid injection, but the symptoms recurred about one month later. Four months later, the patient complained of pain at night and weakness on supination of the forearm and flexion of the elbow. He had no underlying disease, chest symptoms, fever, weight loss, or history of contact with patients suffering from pulmonary tuberculosis.

**Figure 1:**
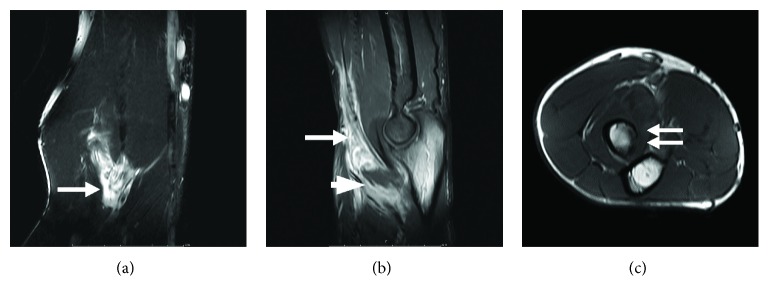
MRI demonstrating a (a) coronal T2-weighted fat-saturated image, (b) sagittal T1-weighted fat-suppressed image with contrast administration, and (c) axial T1-weighted image that showed a disruption of the biceps tendon (arrow in a and b) with ill-defined soft tissue mass (about 2 × 2 cm.). A less enhanced area was noted at the distal part, which was likely to be necrotic or cystic (arrowhead in b). An abnormal marrow signal was detected at the proximal radius with focal cortical erosion at the radial tuberosity (double arrow in c).

Physical examination of the right elbow when patient visited the hospital in Thailand demonstrated swelling at the antecubital fossa, mild tenderness at the distal biceps, and muscle weakness or pain when attempting to supinate the forearm and flex the elbow. All other systemic examinations were normal. There was a high white blood cell count (12,710 cells/mcL); neutrophil count was 72% and lymphocyte count 17%. Erythrocyte sedimentation rate was 17 mm/hr, and C-reactive protein was 6.69 mg/L. Radiography of the right elbow showed swelling at the antecubital fossa, and chest radiographs showed infiltration of the left upper lung. Magnetic resonance images showed disruption of the distal biceps tendon with an associated ill-defined soft tissue mass (about 2 × 2 cm). A less enhanced area was observed at the inferior part, which was likely to be necrotic or cystic. An abnormal marrow signal was detected at the proximal radius with focal cortical erosion at the radial tuberosity (Figure 1).

In this case, we suspected that the patient had disseminated tuberculosis because preoperative chest radiographs demonstrated left upper lung infiltration, which was likely pulmonary tuberculosis, and there was a soft tissue mass at the distal biceps tendon. We performed an open excisional biopsy and debridement using the single-incision anterior approach. The finding was a soft tissue mass involving the distal biceps tendon with complete tendon rupture. There was also a small focal cortical defect at the radial tuberosity. The ruptured distal biceps tendon was debrided. The tendon was repaired to the long-head tendon insertion, which was proximal to the focal defect by about 5 mm, using a TWINFIX Ti 2.8 mm Suture Anchor with one #2 ULTRABRAID Suture (Smith & Nephew Inc.). Antituberculosis chemotherapy started one day after the surgery, following a positive test of the fluid for acid-fast bacilli and a positive polymerase chain reaction for *Mycobacterium tuberculosis*. The patient received a total of 6 months of a rifampin-based regimen, which is recommended for musculoskeletal tuberculosis [3]. The patient initially received isoniazid 300 mg, rifampicin 600 mg, ethambutol 800 mg, and pyrazinamide 1500 mg daily for two months and then reduced to isoniazid and rifampicin for the remaining four months. The elbow was immobilized in a posterior elbow slab with the forearm supinated for four weeks. Mycobacterium culture revealed *Mycobacterium tuberculosis*. Microscopic examination of the soft tissue revealed granulomatous inflammation with multinucleated Langhans giant cells and caseous necrosis.

At the 1-year follow-up, erythrocyte sedimentation rate was 10 mm/hr, and C-reactive protein was 2 mg/L. Motor power of supination and flexion showed grade V and the hook test was negative. This study was approved by the Khon Kaen University Ethics Committee for Human Research (KKUEC) in which the study ID was HE611179.

## 3. Discussion

Tuberculous distal biceps tendon rupture is a rare condition in orthopedics. The incidence of any kind of distal biceps tendon rupture is typically 1.2 per 100,000 patients and is most frequent in individuals from 30 to 39 years of age. The usual causes of rupture are traumatic laceration and spontaneous complete ruptures. Risk factors for tendon rupture are rheumatoid arthritis, gout, ankylosing spondylitis, hemodialysis, hyperparathyroidism, and smoking [4]. Tuberculous tendinitis and bursitis are also a cause of tendon rupture [5].

In this case, the patient was immunocompetent: he had no HIV infection, took no immunosuppressive drugs, was not diabetic, and had a normal complete blood count. The patient had no signs or symptoms specific to musculoskeletal tuberculosis and did not show any symptoms of pulmonary tuberculosis.

Chest radiography shows pulmonary disease in about 50% of cases with osteoarticular tuberculosis, but active pulmonary disease is uncommon [6]. Magnetic resonance imaging was the imaging modality of choice for diagnosis of musculoskeletal tuberculosis because it permits assessment of bony material destruction and identification of soft tissue extension [7].

Laboratory tests, such as erythrocyte sedimentation rate, C-reactive protein, tuberculin skin test, and polymerase chain reaction for *Mycobacterium tuberculosis*, can help the surgeon to detect tuberculosis quickly [1, 2, 5, 8].

Good functional outcomes and low recurrence rates have been reported for musculoskeletal tuberculosis of the hand, with or without tendon rupture, after surgical treatment combined with antituberculosis multidrug therapy. A 6- to 9-month rifampicin-based regimen is recommended for treatment of musculoskeletal tuberculosis [3]. Authors have mentioned that delayed diagnosis and treatment of this condition could be a cause of tendon rupture [5]. In this case, we suspected the diagnosis was disseminated tuberculosis with distal biceps tendon rupture. Therefore, we chose excisional biopsy, anatomic surgical repair, and antituberculosis chemotherapy. The functional outcome for this patient after treatment was excellent.

In conclusion, we demonstrated an immunocompetent patient with the rare manifestation of extraspinal musculoskeletal tuberculosis. The result after treatment was excellent. However, early diagnosis is essential to prevent late complications from tuberculous tendinitis. Therefore, we suggest tests such as erythrocyte sedimentation rate, C-reactive protein, and imaging studies in patients with chronic elbow pain, especially in a population with a high prevalence of tuberculosis.
